# Supplementary material for: Growth in early infancy drives optimal brain functional connectivity which predicts cognitive flexibility in later childhood
Source: bioRxiv. 2025 Mar 17:2024.01.02.573930. Originally published 2024 Jan 3. Preprint. [Version 2] doi: 10.1101/2024.01.02.573930 (PMC10802370; doi:10.1101/2024.01.02.573930)
Supplement: Supplement 1 [file NIHPP2024.01.02.573930V2-supplement-1.pdf]

bioRxiv preprint doi: https://doi.org/10.1101/2024.01.02.573930; this version posted March 17, 2025. The copyright holder for this preprint (which was not certified by peer review) is the author/funder, who has granted bioRxiv a license to display the preprint in perpetuity. It is made available under aCC-BY 4.0 International license.

Supplementary Materials for

Growth in early infancy drives optimal brain functional connectivity which predicts cognitive flexibility in later childhood

Chiara Bulgarelli[1,2], Anna Blasi[2], Samantha McCann[3,4], Bosiljka Milosavljevic[5,6], Giulia Ghillia[3], Ebrima Mbye[4], Ebou Touray[4], Tijan Fadera[4], Lena Acolatse[4,8], Sophie E. Moore[3,4], Sarah Lloyd-Fox[5], Clare E. Elwell[2], Adam T. Eggebrecht[9] and the BRIGHT Study Team[10]

[1]Centre for Brain and Cognitive Development, Birkbeck, University of London, UK.
[2]Department of Medical Physics and Biomedical Engineering, University College London, UK.
[3]Department of Women and Children's Health, King's College London, UK.
[4]Medical Research Council Unit The Gambia at the London School of Hygiene and Tropical Medicine, The Gambia.
[5]Department of Psychology, University of Cambridge, UK.
[6]School of Biological and Experimental Psychology, Queen Mary University of London, UK.
[7]Department of Women and Children's Health, University of Liverpool, UK.
[8]Nutrition Innovation Centre for Food and Health, School of Biomedical Sciences, Ulster University, Ireland.
9Mallinckrodt Institute of Radiology, Washington University School of Medicine in St. Louis, USA.
[10]The BRIGHT team are (in alphabetic order): Muhammed Ceesay, Kassa Kora, Fabakary Njai, Andrew Prentice, Mariama Saidykhan.

This PDF file includes:

Supplementary Results:
Linear mixed model results showing the developmental trajectories of functional connectivity in Gambian infants over the first 2 years of life age (fNIRS pre-processing without global signal regression) 2

Supplementary Figures:
Figure SI1 – Schematic representation of the fNIRS array and the functional connections tested 3
Figure SI2 - Linear mixed models result showing FC that displayed a statistically significant change with age (fNIRS pre-processing without global signal regression) 4
Figure SI3 – An example scatterplot of the association between ΔWLZ and FC at 24 months 5
Figure SI4 – fNIRS preprocessing steps 6
Figure SI5 – The effect of different thresholds for GVTD for motion detecion and minium valid data after pre-processing on data inclusion 7
Figure SI 6 – Strength of correlation between FC in the first and last portion of data 8

Supplementary Tables:
Table SI1 - FC that significantly changed with age (fNIRS pre-processing without global signal regression) 9
Table SI2 - Characteristics of included and excluded participants and seconds of data included in the analyses at each age 10

bioRxiv preprint doi: https://doi.org/10.1101/2024.01.02.573930; this version posted March 17, 2025. The copyright holder for this preprint (which was not certified by peer review) is the author/funder, who has granted bioRxiv a license to display the preprint in perpetuity. It is made available under aCC-BY 4.0 International license.

Supplementary Results

Linear mixed model results showing the developmental trajectories of functional connectivity in Gambian infants over the first 2 years of life age (fNIRS pre-processing without global signal regression)

To rule out that the global signal regression (GSR) performed as part of our fNIRS preprocessing might had an impact on our results, we re-processed the data without this step. All the other steps were kept the same. Hereafter, we reperformed the linear mixed models (LMM) on all the possible 21 interhemispheric homotopic, intrahemispheric, fronto-posterior, and crossed connections.

Results on the Fisher-z transformed correlation coefficients (z-RHO scores) on the oxygenated haemoglobin (HbO_2) showed that left ($F=10.4$, $p<0.001$) and right fronto-middle ($F=4.82$, $p<0.001$) FC increased with age. Results on the Fisher-z transformed correlation coefficients (z-RHO scores) on the deoxygenated haemoglobin (HHb) showed that showed that frontal interhemispheric FC decreased with age ($F=3.5$, $p<0.002$) (**Table SI1** and **Figure SI2**).

Supplementary Figures

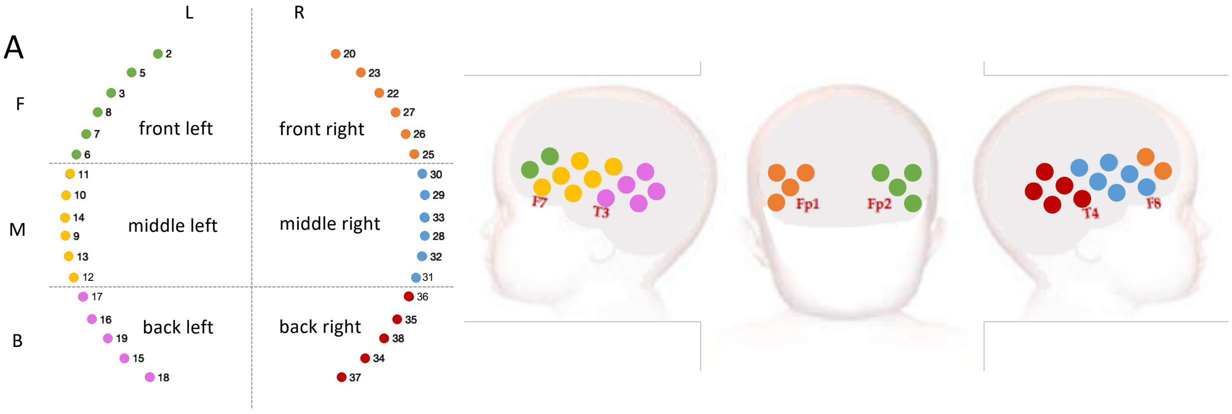

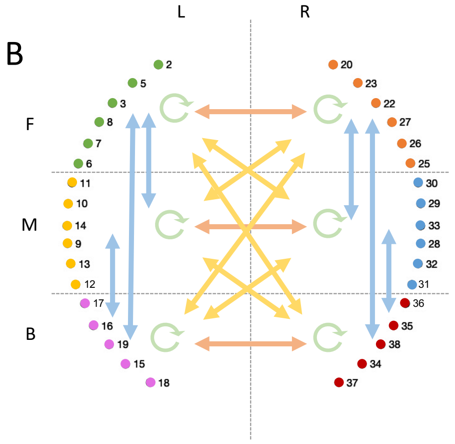

	Left Front	Right Front	Middle Left	Right Middle	Left Back	Right Back
Left Front	Left Front Intra	Front Inter	Left Fronto-Middle	Cross Left Front – middle right	Left Fronto-Back	Cross Left Front – Right Back
Right Front		Right Front Intra	Cross Right Front – Left Middle	Right Fronto-Middle	Cross Right Front – Left Back	Right Fronto-Back
Left Middle			Left Middle Intra	Middle Inter	Left Middle-Back	Cross Left Middle – Right Back
Right Middle				Right Middle Intra	Cross Right Middle – Left Back	Right Middle-Back
Left Back					Left Back Intra	Back Inter
Right Back						Right Back Intra

Figure SI1. Schematic representation of the fNIRS array and the functional connections tested. (**A**) Each dot represents a channel, colours on the left plot corresponds to colours on the right on the baby's head. 6 sections. (**B**) The 21 connections tested in the linear mixed models. Interhemispheric homotopic connections are in orange (connecting the same regions between hemispheres, i.e., front left with front right), intrahemispheric connections are in green (correlations of channels belonging to the same region), fronto-posterior are in blue (connecting front and middle, middle and back, and front and back regions of the same hemisphere), and crossing interhemispheric connections (interhemispheric non-homotopic, connecting the front and middle, middle and back, and front and back regions of the two hemispheres) are in yellow.

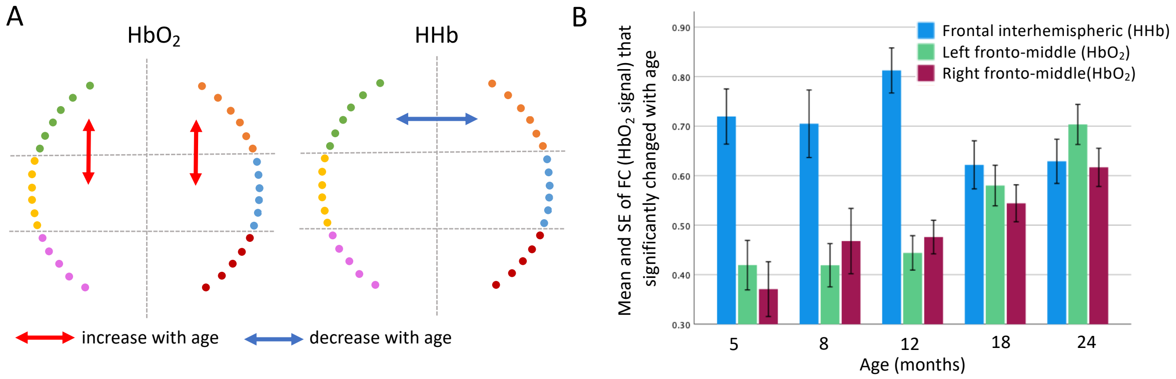

Figure SI2. Linear mixed models result showing FC that displayed a statistically significant change with age. (**A**) Results of the linear mixed model, blue indicates connections that decreased with age, red indicates connections that increase with age. (**B**) Mean and SE of the functional connections that changed with age. Error bars are 1 SE.

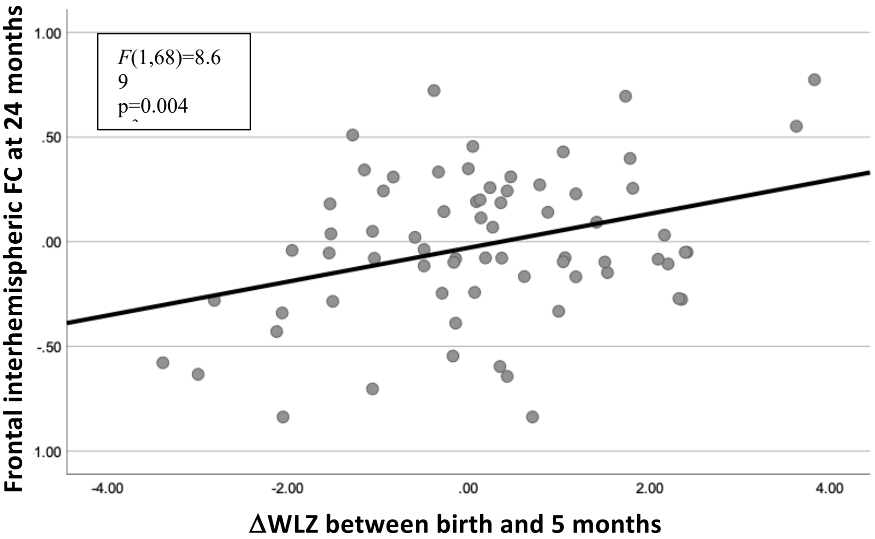

Figure SI3. An example scatterplot of the association between ΔWLZ and FC at 24 months. Scatterplot of the association between ΔWLZ between birth and 5 months and frontal interhemispheric connectivity at 24 months. The black line represents the line of best fit.

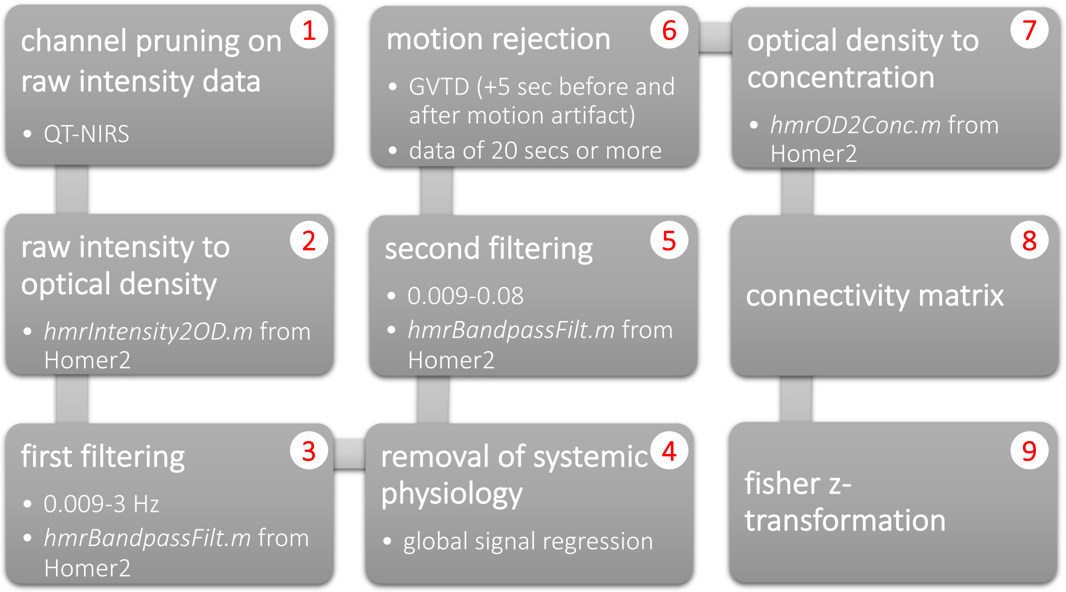

Figure SI4. fNIRS preprocessing steps. The column "infants included in the analyses" in Table 3 refers to those participants whose data survived these preprocessing steps.

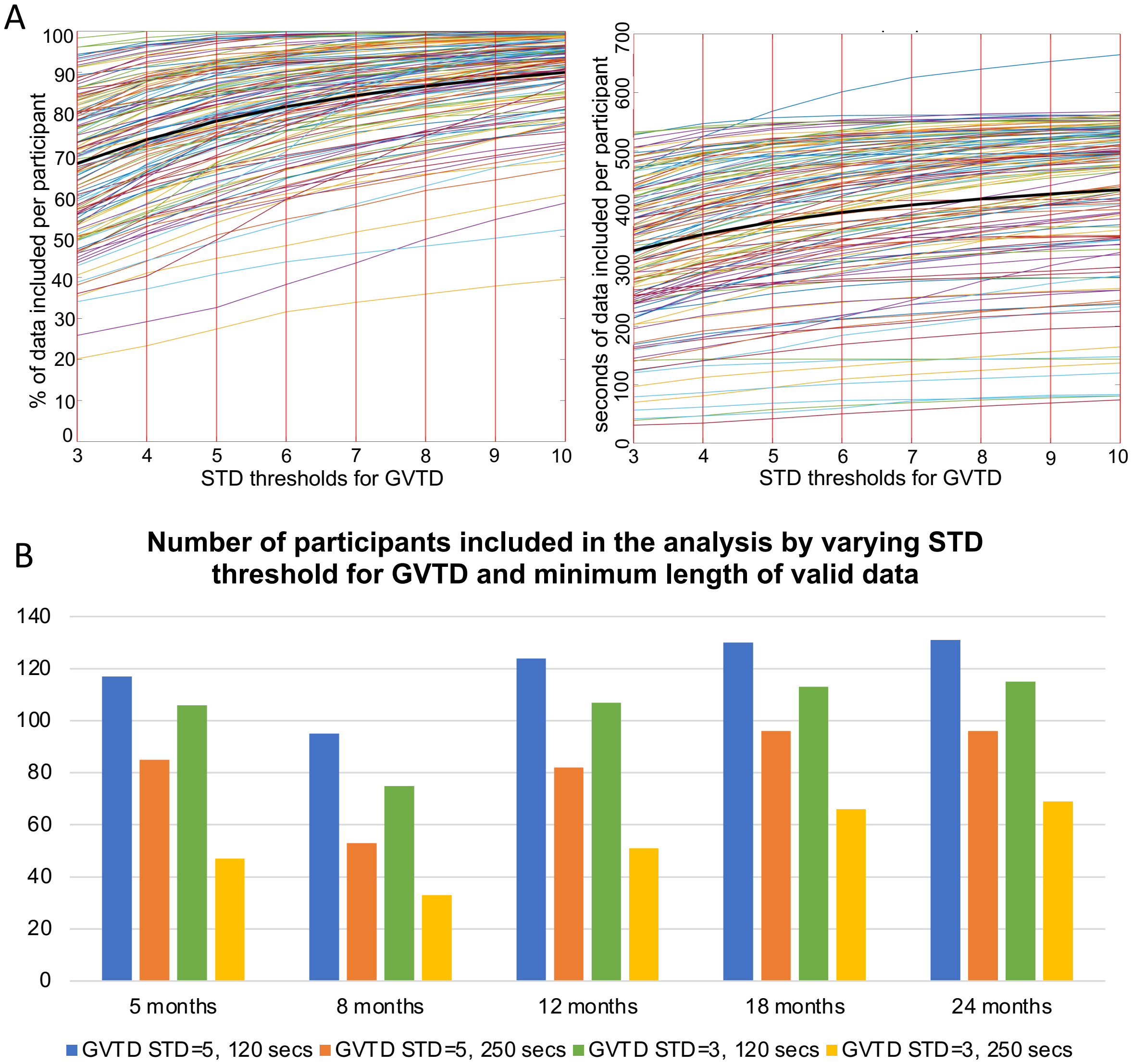

Figure SI5. The effect of different thresholds for GVTD for motion detection and minimum valid data after pre-processing on data inclusion. (A) Percentage of data included (left) and seconds of data included (right) per participant. Each line represents an infant, the black line represents the mean value. These graphs are reported from the 12 months sample as example. (B) Number of infants included in the LMM by varying STD threshold for GVTD and minimum length of valid data at different ages.

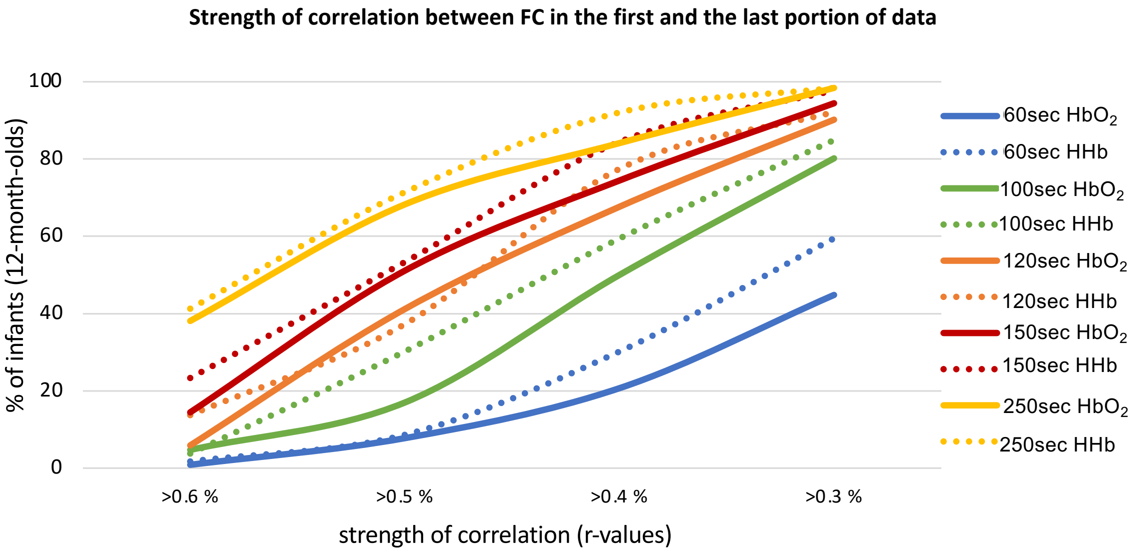

Figure SI6. **Strength of correlation between FC in the first and last portion of data**. This graph is reported from the age 12 month sample as example.

Supplementary Tables

Table SI1. FC that significantly changed with age (fNIRS pre-processing without global signal regression). Results are displayed in terms of estimated betas, standard errors and p values.

**FC**	***F***	***p***	**Baseline (5 months) Betas (SE), *p***	**5-8 change Betas (SE), *p***	**5-12 change Betas (SE), *p***	**5-18 change Betas (SE), *p***	**5-24 change Betas (SE), *p***
**HbO_2**							
Left fronto-middle	10.4	<0.001	-0.55 (0.29), <0.063	0.05 (0.05), 0.239	0.07 (0.04), 0.125	0.16 (0.04), <0.001	0.26 (0.04), <0.001
Right fronto-middle	4.82	<0.001	-0.24 (0.28), 0.398	0.03 (0.05), 0.535	0.05 (0.04), 0.246	0.08 (0.04), 0.051	0.18 (0.04), <0.001
**HHb**							
Frontal interhemispheric	3.5	0.002	0.18 (0.33) 0.591	-0.07 (0.06), 0.260	-0.07 (0.05), 0.214	-0.17 (0.05), 0.002	-0.17 (0.05), 0.002

Table SI2. Characteristics of included and excluded participants and seconds of data included in the analyses at each age. WD=withdrawn, D=deceased, MV=missed visit, DD=developmental delay, NIRS not undertaken = the participant was assessed but did not want or could not perform the NIRS assessments, FC not undertaken = the participant was assessed with other NIRS task, but not FC, Fussed out = the participant wore the headband and the FC acquisition had started but the participant showed signs of fussiness soon after the start of the acquisition, MP=missing pictures of the headband placement, EM=missing event markers, TI=technical issues during the NIRS testing session. The proportion of children included in the analysis was computed based on the infants with FC data.

Age	N - %	Not tested				NIRS not undertaken	FC not undertaken	**Infants with FC data**	Fussed out	Experimental errors			Headband Placement	Too many channels excluded	Not enough data after pre-processing	**Infants included in the analyses**	**Seconds of data included in the analyses (mean±SD)**	**Inclusion rate (from the 204 infants recruited)**
		WD	D	MV	DD					MP	EM	TI						
**5 months**	N	2	1	2	3	10	7	**179**	16	11	3	3	7	5	47	**87**	382.68±92.78	42%
	%	0.98	0.49	0.98	1.47	4.90	3.43	**87.74**	8.93	5.39	1.67	1.67	5.58	2.79	26.25	**48.6**		
**8 months**	N	7	1	5	3	18	14	**156**	7	6	0	1	23	6	60	**53**	372.93±80.66	25%
	%	3.43	0.49	2.45	1.47	8.82	6.86	**76.47**	4.48	3.84	0	0.64	14.7	3.84	38.46	**33.97**		
**12 months**	N	9	1	4	3	17	13	**157**	4	1	0	2	10	2	56	**82**	372.93±80.66	40%
	%	4.41	0.49	1.96	1.47	8.33	6.37	**76.96**	2.54	0.63	0	1.27	6.36	1.27	35.66	**52.22**		
**18 months**	N	8	1	15	3	12	5	**160**	4	0	0	1	16	4	38	**97**	388.85±81.63	47%
	%	3.92	0.49	7.35	1.47	5.88	2.45	**78.43**	2.5	0	0	0.62	10	2.5	23.75	**60.62**		
**24 months**	N	13	1	29	1	3	4	**153**	0	2	0	0	6	4	45	**96**	399.56±79.86	47%
	%	6.37	0.49	14.2	0.49	1.47	1.96	**75**	0	1.30	0	0	3.92	2.61	29.41	**62.74**		
